# The role of HNF4α in adenocarcinoma

**DOI:** 10.1042/BST20253085

**Published:** 2026-04-02

**Authors:** Headtlove Essel Dadzie, Eric L. Snyder

**Affiliations:** 1 Huntsman Cancer Institute, University of Utah, Salt Lake City, UT, U.S.A.; 2 Department of Oncological Sciences, University of Utah, Salt Lake City, UT, U.S.A.; 3 Department of Pathology, University of Utah, Salt Lake City, UT, U.S.A.

**Keywords:** adenocarcinoma, Cancer, HNF4a, lineage plasticity, metabolism, targeted therapy

## Abstract

Hepatocyte nuclear factor 4 alpha (HNF4α) is a conserved nuclear receptor that governs epithelial identity and metabolic homeostasis across endoderm-derived tissues. In cancer, HNF4α can function as either an oncogene or a tumor suppressor. In colorectal and hepatocellular carcinoma, reduced HNF4α activity accompanies loss of differentiation and tumor progression, consistent with tumor-suppressive functions. In contrast, in pancreatic ductal adenocarcinoma, invasive mucinous adenocarcinoma, and other lineage-defined epithelial tumors, HNF4α can also participate in transcriptional programs that sustain malignant identity, metabolic adaptation, and therapeutic resistance. However, these effects are highly context-dependent and do not imply a uniformly oncogenic role in these tumor types. These divergent functions are shaped by isoform usage, chromatin state, epigenetic regulation, metabolic cues, and transcription factor networks. Rather than acting as a classical oncogene or tumor suppressor in all settings, HNF4α is better understood as a context-dependent regulator of lineage state whose activity may either restrain tumor progression or support tumor maintenance. This mini review highlights the molecular mechanisms that shape HNF4α activity, including isoform biology and epigenetic control, and discusses emerging strategies for selectively inhibiting HNF4α in dependency states or restoring its differentiation-promoting functions in tumors where it is lost.

## Introduction

Adenocarcinomas are a diverse group of epithelial malignancies from glandular tissues and encompass tumors from multiple organs, including the lung, gastrointestinal (GI) tract, pancreas, and liver [1]. These cancers represent a major clinical challenge because of their marked heterogeneity, variable differentiation states, and capacity for lineage plasticity. Well-differentiated tumors retain glandular morphology and are often less aggressive, whereas poorly differentiated adenocarcinomas are more likely to exhibit increased plasticity, metastatic potential, and therapeutic resistance [2–4].

Standard treatment strategies such as surgery, chemotherapy, and targeted therapies can be effective in early-stage disease [5,6]. However, advanced adenocarcinomas frequently develop resistance due to tumor heterogeneity, genetic mutations, and adaptive changes in the tumor microenvironment [7,8]. This persistent therapeutic failure underscores the need for approaches that address the lineage and state-specific mechanisms driving tumor progression. In this context, lineage-specific transcription factors are critical regulators of epithelial identity and differentiation, and their dysregulation can disrupt tissue homeostasis and promote tumorigenesis through aberrant plasticity and uncontrolled proliferation [9].

Nuclear receptors (NR) are a specialized class of transcription factors that function primarily as ligand-dependent regulators of gene expression and play essential roles in cell fate determination, metabolism, and tissue homeostasis [10,11]. Hepatocyte nuclear factor 4 alpha (HNF4α) is a conserved orphan NR that regulates epithelial integrity and metabolic homeostasis across multiple organs [12]. Emerging evidence indicates that altered HNF4α expression or activity contributes to adenocarcinoma pathogenesis by shaping tumor differentiation, metabolic programs, and therapeutic susceptibility [13,14]. This review highlights the multifaceted, context-dependent roles of HNF4α across selected epithelial cancers, with emphasis on its potential value as both a biomarker and a therapeutic target.

## HNF4α as a key regulator and therapeutic target

HNF4α is an evolutionarily conserved transcription factor essential for the development and homeostasis of multiple endoderm-derived organs, including the liver, stomach, intestines, and pancreas [15–18]. Across these tissues, it regulates epithelial differentiation, metabolic programs, and tissue-specific gene expression [19]. In the liver, HNF4α orchestrates critical metabolic functions such as glycolysis, glucose transport, and lipid metabolism [15]. Variants in the HNF4A gene have been linked to metabolic disorders such as maturity-onset diabetes of the young 1 (MODY1) and Crohn’s disease, highlighting its systemic importance [18,20].

Beyond its developmental and metabolic functions, HNF4α has emerged as a key regulator in cancer biology, particularly in epithelial malignancies. By maintaining lineage identity and coordinating metabolic state, HNF4α can preserve differentiation and restrain tumor progression in some contexts. In others, however, its activity supports tumor growth by sustaining lineage-specific transcriptional programs and cooperating with oncogenic signaling [13,14,21]. Context-dependent functions are evident across pancreatic [22,23], colorectal [24], liver [25,26], and gastric cancers [21,27–29], where HNF4α output is shaped by isoform usage, tumor stage, microenvironmental cues, and co-regulatory transcription factor interactions.

Together, these observations indicate that HNF4α functions as a regulator of lineage state whose impact depends on disease context. This flexible biology highlights the therapeutic potential of modulating HNF4α activity, either by inhibiting it in tumors that rely on HNF4α-driven programs or by restoring and stabilizing it where loss of HNF4α contributes to dedifferentiation and aggressive behavior.

## Molecular structure of HNF4α

HNF4A encodes a highly conserved nuclear receptor with a modular architecture that underlies its transcriptional activity [12,30]. Like other nuclear receptors, HNF4α contains a DNA-binding domain (DBD) and a ligand-binding domain (LBD), which together mediate sequence-specific DNA recognition and transcriptional regulation [30]. Unlike many nuclear receptors that heterodimerize with retinoid X receptor (RXR), HNF4α forms stable homodimers through structural features that favor exclusive homodimerization [31–33].

The LBD, spanning residues 137 to 384, binds linoleic acid, the predominant endogenous ligand identified for HNF4α [34]. Although HNF4α is generally considered constitutively active, ligand binding appears to stabilize receptor conformation rather than directly mediating classical ligand-dependent transcriptional activation. Ligand exchange has been observed under certain conditions, but its effects on target gene regulation and cofactor recruitment remain incompletely defined [10,34–36].

HNF4α binds direct repeat 1 (DR1) response elements, typically composed of two AGGTCA half-sites separated by a single nucleotide, in both the promoter and enhancer regions of target genes [37]. Although HNF4α has classically been associated with promoter-proximal regulation, genomic and motif-based studies indicate that it also functions at distal regulatory elements, likely in cooperation with other transcription factors and chromatin-associated regulators [22,38–41]. Its transcriptional output is regulated by two activation domains, AF-1 and AF-2. AF-1 contributes to transcriptional activation independently, whereas AF-2 mediates co-regulator interactions in a largely ligand-independent manner [42,43]. A disordered C-terminal F domain further modulates transcriptional output by influencing interactions with co-activators and repressors, and its effects may vary across splice variants [44].

**Figure 1:**
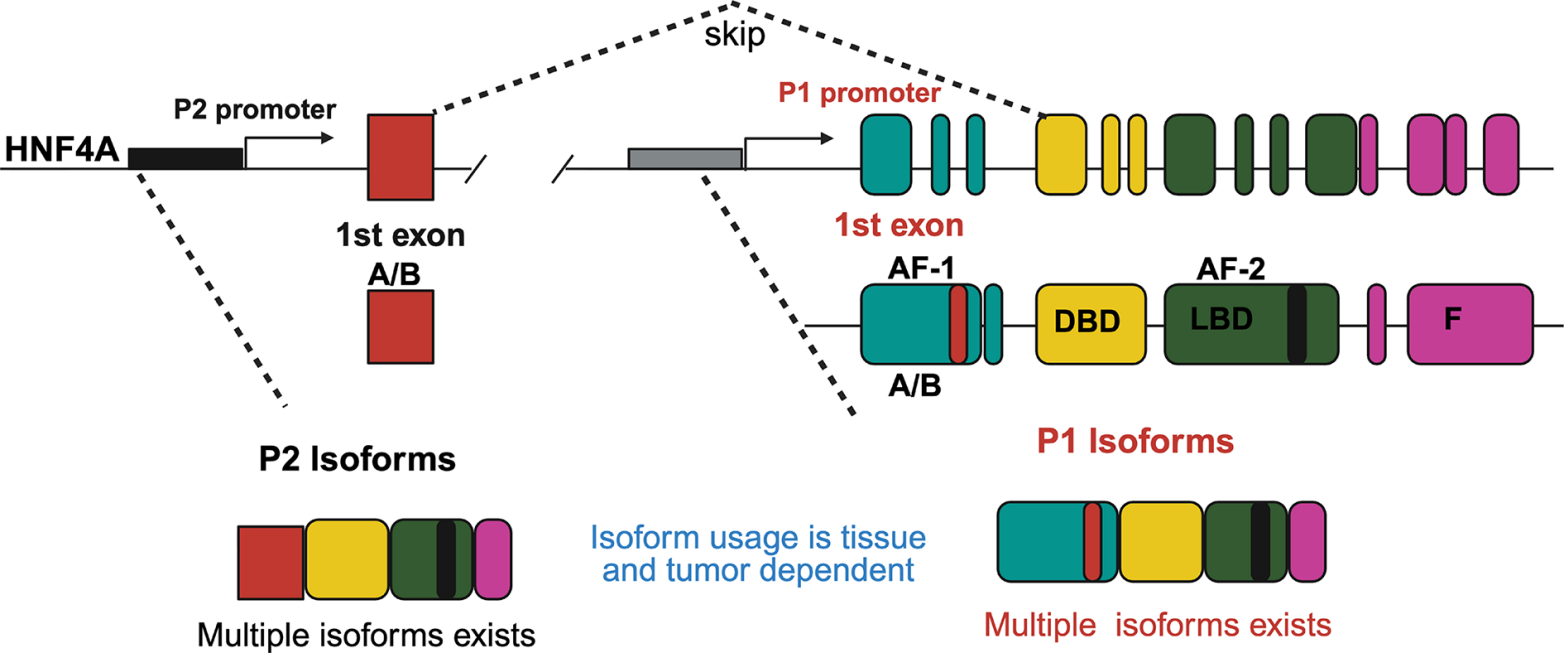
HNF4A gene architecture and isoform diversity Schematic of the human HNF4A locus showing two alternative promoters, P1 and P2, that generate distinct isoforms through differential 5′ exon usage and alternative splicing. Canonical protein domains are indicated, including the DNA-binding domain (DBD), ligand-binding domain (LBD), activation function 1 and 2 domains (AF-1, AF-2), and the F domain. Isoform usage is tissue- and tumor-state dependent.

Alternative promoter usage further expands HNF4α functional diversity. HNF4A generates at least 12 isoforms that are broadly categorized into P1 and P2 classes, which differ in N-terminal structure and transcriptional properties [45]. P1 isoforms, transcribed from the upstream P1 promoter, retain the AF-1 domain and generally exhibit stronger transactivation potential. In contrast, P2 isoforms, transcribed from the downstream P2 promoter, lack AF-1 and therefore display reduced transcriptional potency, as depicted in Figure 1 [18,46].

These isoforms also show distinct tissue distributions. The P1 isoforms predominate in adult liver and kidney, whereas P1 and P2 isoforms are expressed in the pancreas and intestines [47,48]. P2 isoforms are enriched in the stomach, are more prevalent in fetal tissues, and are frequently favored in cancer-associated contexts [47,48]. Notably, shifts from P1- to P2-driven expression have been linked to tumor progression and metabolic dysregulation, highlighting isoform balance as an important determinant of HNF4α function in both normal physiology and disease [22,47–49].

## Regulatory mechanisms shaping HNF4α output

Although HNF4α is not thought to undergo classical endogenous ligand-dependent regulation, its activity is dynamically modulated by post-translational modifications (PTMs) that influence protein stability, DNA binding, subcellular localization, and transactivation. Phosphorylation provides one major layer of regulation [49]. Protein kinase C (PKC)-mediated phosphorylation at Ser78 impairs nuclear retention [50], whereas AMPK-mediated phosphorylation (Ser313 in human HNF4α; Ser304 in rodent HNF4α) reduces DNA binding and promotes protein degradation, linking metabolic signaling to reduced transcriptional output [51,52].

Acetylation provides an additional layer of control, as CBP-mediated acetylation of lysine within the nuclear localization sequence promotes nuclear retention and enhances DNA binding and transactivation [53], while acetylation at Lys458 within the F domain tunes HNF4α transcriptional activity in nutrient-responsive settings [54]. PTM effects can also be isoform selective. For example, Src-mediated phosphorylation of Tyr14, a site-specific to the P1 isoform, is associated with reduced P1 HNF4α abundance and activity in colorectal cancer, illustrating how selective modification can shift isoform balance and functional output [55].

As summarized in Table 1, regulatory mechanisms acting at both the protein and expression levels can bias HNF4α output in a context-dependent manner. In addition to protein PTMs, epigenetic mechanisms that regulate HNF4A expression can converge on similar functional endpoints. For example, PRMT1 and JMJD6 influence HNF4A expression through promoter-associated arginine methylation in alcohol-related liver disease models [56], while promoter DNA methylation has been linked to HNF4A repression in pancreatic cancer [57]. Together, these regulatory layers help explain how HNF4α activity may be maintained in some contexts yet attenuated or lost in others, contributing to its tumor-suppressive versus tumor-supportive roles across epithelial cancers [58].

**Table 1:** Protein and epigenetic regulatory mechanisms that shape HNF4α output

Modification	Primary enzyme	Target site	Cancer context	Functional outcome	Clinical role	Reference
Phosphorylation	Src Kinase	Tyr14	Colorectal	Reduces P1 HNF4α abundance and activity in an isoform specific manner	Bias toward loss of differentiation programs	[55]
Phosphorylation	AMPK	Ser 304 (rodent)/ Ser313 (human)	Liver metabolism	Reduces dimerization and DNA binding, repressing transcriptional activity	Bias toward reduced HNF4α output	[51,52]
Phosphorylation	PKC	Ser78	HepG2 (Liver derived tumor cells)	Impairs nuclear localization and reduces DNA binding and transactivation; decreases protein stability via proteasome dependent loss	Bias toward reduced HNF4α output	[50]
Acetylation	CBP	NLS lysines	Hepatocyte / epithelial contexts	Promotes nuclear retention and increases DNA binding and transactivation	Bias toward active HNF4α	[53]
Acetylation	Not defined	Lys458 (F domain)	HepG2 / liver - related context	Represses HNF4α transcriptional activity; nutrient responsive	Bias toward decreased HNF4α output	[54]
Arginine methylation* (chromatin)	PRMT1 (opposed by JMJD6)	HNF4A promoter	Alcohol - related liver context	Maintains HNF4A expression via promoter arginine methylation; JMJD6 counteracts this	Bias toward maintaining HNF4α programs	[56]
DNA methylation* (epigenetic)	DNMTs	HNF4A promoter	Pancreatic cancer	Promoter methylation represses HNF4A expression	Bias toward HNF4α loss	[57]

Representative protein PTMs and epigenetic mechanisms* that modulate effective HNF4α activity are summarized, including phosphorylation and acetylation events that alter nuclear localization, DNA binding, transactivation, and stability, as well as chromatin and promoter modifications that regulate HNF4A expression. Together, these regulatory layers can bias HNF4α toward active differentiation-associated programs or toward reduced output and functional loss in a context-dependent manner across tumor types.

## Biological role of HNF4α

### Epithelial differentiation

HNF4α is a central regulator of epithelial differentiation across multiple endoderm-derived organs, including the liver, pancreas, and intestine. In the liver, it maintains hepatocyte identity and supports expression of a large fraction of actively transcribed hepatic genes, underscoring its importance in liver function [59,60]. During development, HNF4α promotes hepatoblast maturation and epithelial organization, whereas its loss disrupts hepatocyte differentiation and liver architecture [59,60]. In the intestine, HNF4α contributes to crypt organization, epithelial lineage specification, and goblet cell maturation. Consistent with these roles, HNF4A dysregulation has been linked to gastrointestinal disease states, including colitis and colorectal cancer, and genetic variation in HNF4A has been associated with ulcerative colitis [61,62].

### Metabolic regulation

HNF4α is a master regulator of hepatic metabolism, coordinating transcriptional programs that govern glucose, lipid, amino acid, and bile acid homeostasis [13,14,63]. It helps maintain metabolic balance across fasting and feeding states by regulating gluconeogenesis and insulin sensitivity [64]. HNF4α also regulates fatty acid oxidation, lipoprotein metabolism, and lipid storage through control of genes involved in lipid handling, including scavenger receptor class B type 1 (SR-B1) [65,66]. In addition, HNF4α influences detoxification and bile acid synthesis by regulating cytochrome P450 enzymes and transporters required for drug clearance and lipid digestion [67]. In cancer, aberrant HNF4α expression may contribute to metabolic reprogramming by uncoupling nutrient utilization from normal differentiation programs, thereby supporting tumor growth and adaptation [12–14].

## HNF4α in cancer biology: oncogenic versus suppressive roles

The role of HNF4α in cancer is complex and context-dependent. As a regulator of epithelial differentiation and lineage fidelity, HNF4α can suppress tumorigenesis by reinforcing metabolic homeostasis, cell adhesion, and epithelial polarity [18]. In several cancer types, loss of HNF4α leads to an undifferentiated, aggressive phenotype [68–70]. In other contexts, however, HNF4α can promote tumor progression by cooperating with oncogenic signaling pathways and sustaining proliferative transcriptional programs [71–73]. This functional duality is a recurring theme across epithelial cancers, where HNF4α activity is shaped by tumor state, microenvironmental cues, and underlying genetic alterations [12,14,74].

## HNF4α in hepatocellular carcinoma

Comprehensive discussions of HNF4α function in the liver, spanning both benign hepatic lesions and malignant disease contexts, have been recently published [75,76]. HNF4α is expressed most abundantly in hepatocytes, where it anchors transcriptional programs required for liver differentiation, metabolic balance, and epithelial identity [12,25,63]. It regulates hepatocyte-defining programs controlling glucose and lipid metabolism, bile acid synthesis, xenobiotic detoxification, and epithelial polarity [12,25,77], and is sufficient to reprogram fibroblasts into functional hepatocyte-like cells, underscoring its role as a master regulator of hepatocyte identity [78,79].

Hepatocellular carcinoma (HCC) is an epithelial tumor derived from hepatocytes and accounts for most primary liver cancers [80], ranking as the sixth most commonly diagnosed cancer and the third leading cause of cancer-related mortality globally [81,82]. Despite arising from terminally differentiated hepatocytes, HCC frequently exhibits profound transcriptional reprogramming, and the role of HNF4α in this process is paradoxical and highly context-dependent, with evidence supporting both tumor-suppressive and tumor-promoting functions [75,83,84].

In several studies, HNF4α has been shown to exert tumor-suppressive functions in HCC, including inhibition of β-catenin signaling, suppression of epithelial-to-mesenchymal transition through miR-29-mediated repression of DNMT3s, and maintenance of hepatocyte differentiation programs [68,85]. Recent work further suggests that HNF4α can switch between tumor-suppressive and tumor-promoting roles depending on AMPK pathway activity, directly linking cellular metabolic state to its divergent functions in HCC [26].

Although hepatocellular adenomas are benign lesions that rarely progress to HCC, molecular subtypes such as HNF1A-inactivated adenomas underscore the importance of hepatocyte lineage transcriptional networks in maintaining metabolic identity [86,87]. Disruption of this broader hepatocyte differentiation axis is associated with diffuse steatosis and altered transcriptional programs, highlighting how loss of lineage control can reshape metabolic state even in benign hepatic lesions [88,89]. While these lesions are driven primarily by HNF1A alteration, they illustrate a broader principle relevant to HNF4α biology: destabilization of hepatocyte lineage-specifying transcriptional networks can reshape metabolic state and erode differentiated identity.

Collectively, these studies suggest that HNF4α primarily functions to stabilize hepatocyte lineage identity, with its impact on tumor progression determined by metabolic cues and the transcriptional landscape in which it operates. In HCC, disruption of HNF4α-centered regulatory networks promotes loss of differentiated programs and increased plasticity, whereas maintenance or rewiring of these networks can support tumor growth in metabolically permissive contexts.

## HNF4α in gastrointestinal adenocarcinomas

Gastrointestinal adenocarcinomas (GIACs), including tumors of the stomach, small intestine, colon, and rectum, comprise a heterogeneous group of epithelial malignancies that nevertheless share recurrent dependence on lineage-regulatory transcriptional programs [21]. HNF4α is a core epithelial regulator in the normal gastrointestinal tract and is aberrantly activated in subsets of GI cancers, in part through copy number gain and epigenetic remodeling that enhance HNF4A transcriptional output [21]. Integrative analyses of TCGA and CCLE datasets further show that *HNF4A* is overexpressed across GIACs, where it co-regulates transcriptional programs with factors such as *GATA4/6, ELF3*, and *KLF5*, underscoring its conserved role in tumor maintenance [21].

Gastric cancer (GC) is the fifth most common cancer and the fourth leading cause of cancer-related death globally [90]. Major risk factors include *Helicobacter pylori* infection, diet, smoking, and genetic predisposition [91]. Despite treatment advances, the five-year survival rate remains low, particularly in advanced disease [92]. In gastric cancer, HNF4A is broadly expressed across molecular subtypes and functions as a lineage-defining transcription factor [21,93]. It contributes to maintenance of super-enhancer landscapes and integrates metabolic and oncogenic signaling networks. Mechanistically, HNF4α modulates AMPK–Wnt cross-talk and acts downstream of transcription factors such as KLF5 and GATA4/6 [21,93,94]. In addition, HNF4α-driven metabolic reprogramming can support tumorigenesis through IDH1-dependent regulation of α-ketoglutarate metabolism, linking lineage control to a potentially targetable metabolic dependency [29]. Together, these findings position HNF4α as a candidate lineage-dependent vulnerability in gastric cancer.

In colorectal cancer (CRC), which is the third most commonly diagnosed cancer, HNF4α function is strongly isoform-dependent [95]. P1-HNF4α, primarily expressed in differentiated colonic epithelial cells, has been associated with tumor suppression, while P2-HNF4α, enriched in proliferative compartments, is linked to tumor progression [24,70,71]. At the same time, elevated HNF4α expression in CRC can support oncogenic signaling through cross-talk with Wnt/β-catenin and AP-1 pathways [70]. Experimental models further demonstrate distinct isoform effects in colitis-associated tumorigenesis: P1-HNF4α protects against colitis and reduces tumor burden, whereas P2-HNF4α promotes inflammation-driven tumorigenesis, in part through induction of RELMβ [71]. More recent studies have uncovered a feedback loop between HNF4α and Wnt/β-catenin signaling in CRC progression, suggesting that HNF4α responds to oncogenic cues and actively shapes tumor biology [96].

Across GIACs, the clinical significance of HNF4α is therefore context and isoform-dependent. In gastric cancer, HNF4α more often behaves as a lineage-maintaining dependency linked to transcriptional and metabolic fitness, whereas in colorectal cancer its function can diverge substantially between P1 and P2 isoforms. These distinctions will be important for determining whether HNF4α should be inhibited, stratified by isoform and tumor state, or used primarily as a biomarker in GI malignancies.

## HNF4α in pancreatic ductal adenocarcinoma

Pancreatic ductal adenocarcinoma (PDAC) is one of the most lethal malignancies, characterized by aggressive biology and limited treatment options. HNF4α plays a complex role in the pancreas by maintaining epithelial differentiation and metabolic homeostasis. During pancreatic development and endocrine maturation, HNF4α regulates genes essential for islet cell function, and pathogenic HNF4A variants are linked to MODY1 [20,97].

PDAC can be broadly divided into two major transcriptional programs that reflect distinct epithelial identities. The classical subtype retains a differentiated ductal phenotype and is consistently associated with more favorable clinical behavior. In contrast, the basal-like or squamous subtype shows coordinated silencing of core endodermal lineage regulators, including *HNF4A*, *HNF1A*, and *GATA6*, together with extensive metabolic rewiring and a more aggressive clinical course [98–100]. These subtype differences raised the question of whether HNF4α is merely a marker of the classical state or an active determinant of lineage identity. Camolotto and colleagues showed that HNF4α helps maintain the classical phenotype by activating classical-state transcriptional programs while repressing a subset of basal-like genes. Although HNF4α represses a subset of basal-like genes, including *Six1* and *Six4*, *Hnf4a* deletion in mouse PDAC organoids was insufficient to fully induce the basal-like phenotype, indicating that additional factors are required for this transition [23]. Consistent with this, *HNF4A* down-regulation is a hallmark of the basal-like subtype, suggesting that loss of HNF4α is an early event in subtype transition [100]. Together, these findings support a model in which HNF4α functions as an important regulator of classical-state identity, but not as the sole determinant of basal-like switching.

Beyond transcriptional identity, loss of HNF4α also promotes metabolic reprogramming. In PDAC models, loss of HNF4α and GATA6 defines therapeutically actionable squamous-like states, including dependence on GSK3β and enhanced glycolytic programs, with selective sensitivity to GSK3β inhibition in subsets of squamous tumors [101]. However, rapid emergence of drug resistance in some patient-derived squamous models suggests that compensatory metabolic pathways or epigenetic plasticity can blunt this vulnerability [101]. These findings underscore the need to define resistance mechanisms if lineage-directed therapies are to be deployed effectively.

The P1 and P2 isoforms of HNF4α perform distinct roles in PDAC biology. Our recent work shows that HNF4α-positive PDAC tumors consistently express the P2 isoform, while the expression of the P1 isoform varies [22]. Functional, transcriptomic, and epigenetic analyses further indicate that P1 isoforms act as stronger transcriptional regulators than P2 isoforms, with more robust binding at HNF4α target genes and greater transactivation potential. Despite sharing a common DBD, however, P1 isoforms appear less compatible with tumor growth than P2 isoforms, suggesting a more restrictive role in PDAC progression. At the same time, studies in human PDAC cell lines indicate that P1 and P2 isoforms retain substantial functional redundancy because of overlapping genomic binding sites [22]. Together, these findings suggest that isoform balance, rather than absolute HNF4α expression alone, is an important determinant of PDAC phenotype.

More broadly, HNF4α regulates PDAC subtype identity as part of a hierarchical transcription factor network that includes GATA6 and HNF1A. Loss of GATA6 is necessary but not sufficient to induce the basal phenotype, whereas combined loss of GATA6, HNF4α, and HNF1A more effectively drives lineage switching [101,102]. Patient-derived samples also suggest that *GATA6* down-regulation often precedes HNF4α loss during progression toward the basal-like state [101]. Sequential loss of these lineage specifiers is associated with broad epigenomic remodeling, particularly at super-enhancers, enabling ΔNp63-driven transcriptional programs that reinforce basal identity [103,104].

## HNF4α in lung adenocarcinoma

Lung cancer remains the leading cause of cancer-related mortality and lung adenocarcinoma (LUAD) is the most common subtype of lung cancer [95]. LUAD can be stratified in part by expression of the lineage specifier NKX2-1, with NKX2-1-positive tumors generally associated with better prognosis [105,106]. Although HNF4α is not typically expressed in lung tissue during early endodermal development or adulthood, it is notably expressed in specific LUAD subtypes [107,108].

Invasive mucinous adenocarcinoma (IMA) of the lung, a distinct NKX2-1 negative subtype accounting for approximately 5% of LUAD cases, exhibits high levels of HNF4α expression, with up to 95% of IMA tumors expressing HNF4α, making it a distinguishing marker relative to other gastric-related genes [109,110]. In mouse models of IMA, deletion of *Hnf4a* in the context of NKX2-1 loss significantly impaired tumor initiation, supporting its role as a potential master regulator of the cellular identity switch observed in IMA [111]. In a recent bioRxiv preprint that has not yet been peer reviewed, spatial transcriptomic analyses further reinforce HNF4α’s pivotal role in IMA. Spatial transcriptomic profiling of human IMA samples identified distinct mucinous tumor cell clusters expressing HNF4A, GKN1, and FOXA3, further distinguishing IMA from lung adenocarcinoma with signet ring cell features (SRCC) [112]. In that non-peer-reviewed study, pharmacologic inhibition of HNF4α with BI6015 suppressed tumor cell growth and that HNF4α induces *MUC3A/B* and *TM4SF4*, supporting a potential therapeutic vulnerability and reinforcing its role in the mucinous phenotype of IMA [112]. In human IMA cell lines, the P2 isoform of HNF4α was recently identified as a key driver of tumor growth and metastasis. Here, the authors showed that HNF4α up-regulates the lncRNA BC200, stabilizing mRNAs for oncogenic proteins via FMR1, creating a feed-forward loop sustaining HNF4α expression. Notably, mycophenolic acid (MPA), the active metabolite of mycophenolate mofetil (MMF), was identified as an HNF4α antagonist with anti-tumor effects in IMA in the present study [113].

Building on these observations, our recent work establishes HNF4α as a central regulator of IMA biology across genetically engineered mouse models, mouse organoids, and patient-derived organoids [114]. We showed that HNF4α promotes tumor growth by driving a gastric differentiation program, particularly the pit-cell lineage, through direct regulation of gastric promoters and enhancers. Loss of HNF4α disrupted this lineage state, permitted *de novo* FoxA1/2 chromatin redistribution, and promoted alternate transcriptional programs, including neuronal and liver-like states. Functionally, *Hnf4a* deletion sensitized IMA to the KRAS^G12D^ inhibitors *in vitro* and *in vivo*, linking HNF4α-dependent lineage maintenance to both tumor fitness and therapeutic resistance [114]. Collectively, these findings establish HNF4α as a critical driver of IMA biology, modulating both gastric differentiation and resistance to KRAS inhibitors.

A hybrid identity subtype of LUAD characterized by co-activation of pulmonary and gastric differentiation programs has also been identified [115,116]. Single-cell RNA sequencing and protein analyses reveal that these hybrid cells co-express NKX2-1 and HNF4α, with the latter robustly marking the gastric-like phenotype [115,116]. Consistent with these observations, HNF4α expression in LUAD has also been associated with distinct clinicopathological and genetic features, further supporting its value as a marker of lineage-divergent tumor states [117]. Building on these observations, our recent work indicates that HNF4α promotes a gastrointestinal/liver-like state by activating its canonical target genes whilst dampening NKX2-1 binding, thereby enabling simultaneous expression of pulmonary and gastric lineage programs in LUAD cells [118]. In our models, loss of HNF4α increases sensitivity to KRAS^G12D^ inhibition, highlighting its role in mediating therapeutic resistance in this LUAD subtype [118]. Together, these findings underscore the role of HNF4α in regulating hybrid lineage identity and support its potential as a therapeutic target in both IMA and LUAD with mixed differentiation.

## HNF4α in pulmonary large cell neuroendocrine carcinomas

Pulmonary large cell neuroendocrine carcinomas (LCNECs) account for 2–3% of lung cancers and represent a distinct subgroup within high-grade neuroendocrine lung tumors, alongside small cell lung cancer (SCLC) and pulmonary carcinoids [119]. Because LCNECs lack a standardized treatment strategy, defining their molecular subtypes and lineage dependencies remains an important clinical priority. LCNECs can be divided into two subtypes. Type 1 LCNECs harbor STK11/KEAP1 alterations and express gastrointestinal transcription factors such as HNF4A, HNF1A, and RFX6. In contrast, Type 2 LCNECs harbor RB1 alterations and exhibit low neuroendocrine marker expression but display high NOTCH pathway activity and enhanced immune responses [120].

A broader pan-cancer classification of neuroendocrine tumors has identified a corresponding subtype, termed Subtype H NECs, characterized by up-regulation of HNF-family transcription factor targets [121]. Functional studies in representative human subtype H NEC cell lines showed that *HNF4A* knockout induces extensive transcriptomic reprogramming, including loss of gastroenteropancreatic (GEP) gene signatures and shifts in neuroendocrine fate regulators, supporting HNF4α as a key determinant of lineage identity in this group [121]. In addition to defining lineage identity, subtype H NECs retain functional RB protein and exhibit striking resistance to standard NEC chemotherapies, including carboplatin, etoposide, and their combination, compared with other NEC subtypes [121]. This resistance may reflect the combined effects of intact RB signaling, KEAP1–NRF2-associated metabolic reprogramming, and xenobiotic metabolism linked to gastroenteropancreatic differentiation [121,122].

Together, these findings position HNF4α-positive LCNEC as a distinct, chemo-resistant lineage state within the broader neuroendocrine lung cancer spectrum. However, important questions remain regarding how HNF4α interfaces with neuroendocrine differentiation programs and whether this lineage dependency can be therapeutically exploited in LCNEC and related HNF4α-positive lung neuroendocrine tumors [122].

## HNF4α as a context-dependent therapeutic target

HNF4α functions in a highly context-dependent manner across epithelial cancers, acting as either a tumor suppressor or a driver of malignant programs depending on tissue lineage, isoform usage, disease stage, and cellular state [12,14,74]. Across adenocarcinomas, HNF4α frequently tracks with differentiated glandular programs and can either constrain progression or sustain lineage maintenance depending on the tumor context [49,123]. In subsets of colorectal and gastric adenocarcinoma, reduced HNF4α activity has been linked to loss of differentiation and tumor progression, supporting a tumor suppressive framework in selected settings [27,124]. By contrast, in certain subtypes of LUAD, including the hybrid subtype and IMA, HNF4α supports lineage-specific transcriptional and metabolic programs that maintain tumor fitness and promote therapeutic resistance, indicating an oncogenic dependency in defined settings [114,118].

Importantly, the tractability of nuclear receptors as a therapeutic class [125,126] raises the possibility that HNF4α may also be pharmacologically manipulated. Consistent with this concept, the development of small molecule HNF4α antagonists such as BI6015 and emerging HNF4α agonists [36,127,128] has provided proof of principle that HNF4α-dependent transcriptional programs can be disrupted or activated in cancer models, although these agents have not advanced beyond preclinical use [126]. Together, these observations underscore that HNF4α does not fit neatly into a conventional oncogene or tumor suppressor framework but instead functions as a context-dependent regulator of epithelial tumor state [14].

**Figure 2:**
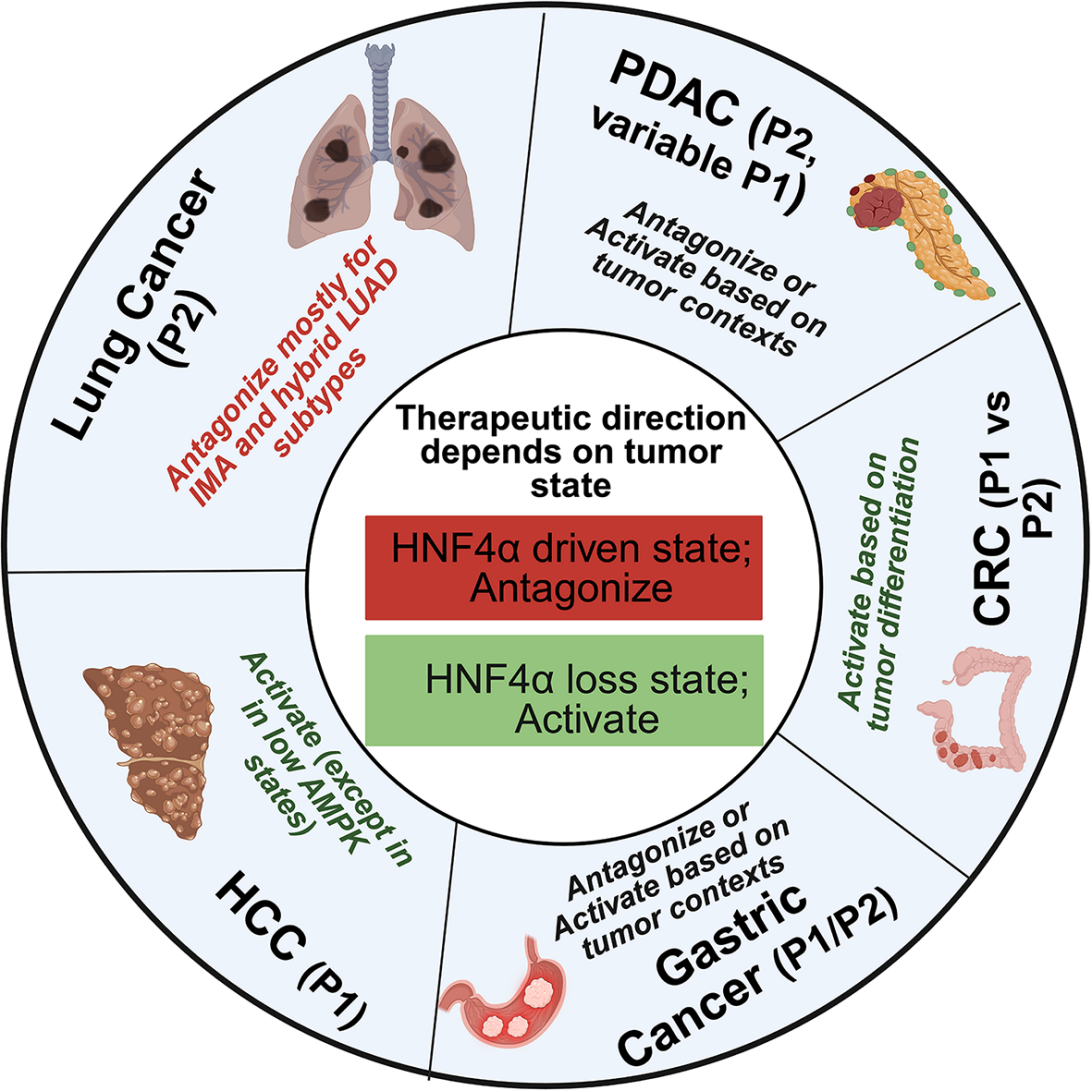
Tumor state-dependent therapeutic direction for HNF4α across cancer types Schematic illustrating that the optimal strategy for targeting HNF4α depends on tumor state. Centre, an HNF4α-driven state is proposed to favor antagonism (red), whereas an HNF4α loss or low activity state is proposed to favor activation (green). Outer ring shows example tumor types with predominant HNF4A promoter usage (P1 versus P2) and context-specific therapeutic directionality.

In tumors that rely on HNF4α activity, inhibition may suppress growth and enhance sensitivity to targeted therapies. Conversely, in contexts where HNF4α is diminished, restoring its function may promote differentiation and limit aggressive behavior [36,129,130] (Figure 2). Emerging evidence linking HNF4α activity to resistance to KRAS^G12D^ inhibition suggests that combined targeting of lineage programs and oncogenic signaling may be a promising strategy, especially in mucinous and hybrid lung adenocarcinoma states [114,118].

## Concluding perspectives and future directions

Although much of the translational evidence for HNF4α comes from glandular lineages, its potential as a therapeutic target has also been discussed in other cancer types, supporting a broader, lineage-based framework [14]. In HCC, HNF4α is closely tied to hepatocyte differentiation and metabolic identity [63], making restoration or stabilization paradigms conceptually distinct from inhibition strategies considered for HNF4α-dependent adenocarcinoma states [130]. Outside glandular cancers, HNF4α-linked programs have been described in subsets of squamous cancers (for example head and neck squamous cell carcinoma) [131], and HNF4α-regulated metabolic axes that modulate drug response have been reported in urothelial bladder cancer [132]. HNF4α-defined transcriptional states are also evident in selected neuroendocrine carcinomas, where they may reflect partial adoption of endodermal-like regulatory programs rather than canonical gland formation [120–122]. Together, these observations reinforce a practical point for drug development: HNF4α should be viewed as a lineage state regulator, such that the therapeutic objective must be defined by the state it stabilizes in a given tumor, whether that entails suppressing an HNF4α-anchored survival program or preserving differentiation to limit plasticity, ideally in conjunction with biomarker-guided patient selection [14].

Moving forward, the development of selective HNF4α modulators will be critical to fully leverage its therapeutic potential, including antagonists for oncogenic contexts and agonists or activators for tumor-suppressive settings [36,133]. Future studies should prioritize defining isoform-specific functions, co-regulatory networks, and chromatin dependencies across tumor types using integrated multi-omics and functional models. As understanding of HNF4α biology deepens, it may be repositioned from a passive lineage marker to an actionable determinant of tumor state. Ultimately, integrating HNF4α-centered strategies into biomarker-guided therapeutic frameworks may improve outcomes across diverse epithelial cancers.

## Perspectives

- **Importance of the field:** Understanding how transcriptional regulators govern lineage stability and plasticity is central to explaining phenotypic diversity, tumor evolution, and therapeutic vulnerability across adenocarcinomas.
- **Current thinking:** HNF4α is increasingly viewed as a context-dependent regulator of epithelial tumor state, operating within transcriptional networks shaped by isoform usage, chromatin state, and cellular signaling.
- **Future directions:** Defining how isoform-specific regulation, chromatin context, metabolic signaling, and upstream cues converge to modulate HNF4α activity across differentiation states will be essential for understanding lineage behavior, disease progression, and therapeutic opportunity.

## Open Access

Open access for this article is provided via our Subscribe to Open programme, made possible through the support of our subscribing institutions. Learn more here: https://portlandpress.com/pages/open_access_options_and_prices#conditional.

## Abbreviations

AF-1: Activation Function 1
AF-2: Activation Function 2
AMPK: AMP-activated protein kinase
BC200: Brain cytoplasmic RNA 200
CBP/p300: CREB-binding protein/ E1A-binding protein p300
CCLE: Cancer Cell Line Encyclopedia
ChIP-seq: Chromatin immunoprecipitation sequencing
CRC: Colorectal cancer
DNMTs: DNA methyltransferases
DTPs: Drug-tolerant persister cells
EMT: Epithelial-mesenchymal transition
ERK: Extracellular signal-regulated kinase
FMR1: Fragile X messenger ribonucleoprotein 1
GEMM: Genetically engineered mouse model
GIACs: Gastrointestinal adenocarcinomas
GSK3β: Glycogen synthase kinase 3 beta
HCC: Hepatocellular carcinoma
HNF4α: Hepatocyte nuclear factor 4 alpha
IMA: Invasive mucinous adenocarcinoma
KEAP1: Kelch-like ECH-associated protein 1
KRAS: Kirsten rat sarcoma viral oncogene homolog
LCNEC: Large cell neuroendocrine carcinoma
lncRNA: Long noncoding RNA
LNETs: Lung neuroendocrine tumors
LUAD: Lung adenocarcinoma
MCM2: Minichromosome maintenance complex component 2
MMF: Mycophenolate mofetil
MODY1: Maturity-onset diabetes of the young type 1
MPA: Mycophenolic acid
NECs: Neuroendocrine carcinomas
NRs: Nuclear receptors
NSCLC: Non-small cell lung cancer
PDAC: Pancreatic ductal adenocarcinoma
PDOs: Patient-derived organoids
PRMT1: Protein arginine methyltransferase 1
PTMs: Post-translational modifications
RB1: Retinoblastoma protein 1
RXR: Retinoid X receptor
SCLC: Small cell lung cancer
SRC: SRC proto-oncogene, non-receptor tyrosine kinase
SRCC: Signet ring cell carcinoma
STK11: Serine/threonine kinase 11
TCGA: The Cancer Genome Atlas
Wnt: Wingless-related integration site
